# Quality of life and its predictors in first-episode schizophrenia and individuals at clinical high-risk for psychosis

**DOI:** 10.1186/s12888-023-05303-9

**Published:** 2023-10-31

**Authors:** Zhen Mao, Lu Tian, Yue Sun, Fang Dong, Chuanyue Wang, Qijing Bo

**Affiliations:** 1 The National Clinical Research Center for Mental Disorders & Beijing Key Laboratory of Mental Disorders & Beijing Institute for Brain Disorders Center of Schizophrenia, Beijing Anding Hospital, Capital Medical University, No.5 Ankang Lane, Dewai Avenue, Xicheng District, Beijing, 100088 China; 2 Advanced Innovation Center for Human Brain Protection, Capital Medical University, Beijing, 100069 China

**Keywords:** Clinical high-risk for psychosis, Schizophrenia, First episode schizophrenia, Quality of life

## Abstract

**Background:**

This is a cross-sectional study comparing the degree of subjective quality of life (QOL) impairment and its predictive factors in first-episode schizophrenia (FES) and individuals at clinical high-risk (CHR) for psychosis.

**Methods:**

Seventy-seven FES, 59 CHR, and 64 healthy controls (HC) were included. The QOL of all participants was assessed using the World Health Organization Quality of Life (WHOQOL)-Brief Form (BREF). Psychiatric symptoms of individuals with FES were assessed with the Positive and Negative Syndrome Scale (PANSS), five factors were further identified through factor analysis; for individuals with CHR and HC, the Scale of Prodromal Symptoms (SOPS) was used.

**Results:**

The total and four sub-domain scores of the WHOQOL-BREF in the FES and CHR groups were lower than those of the HC group. The overall and psychological health scores in the CHR group were lowest. In the FES group, after applying Bonferroni’s correction, there is a negative correlation between the total QOL scores and anxiety/depressive symptom scores (*r* = –0.34, *P* = 0.003). The stepwise multiple regression analysis showed that the QOL of both FES and CHR group were negatively affected by anxiety/depressive symptoms and unemployment (*P* < 0.05).

**Conclusions:**

Compared with FES, CHR individuals are more dissatisfied with their QOL. Although diagnostic assessment of FES and CHR relies heavily on positive symptoms, the QOL is more affected by anxiety/depressive symptoms and social functioning.

## Background

Psychiatric clinicians aim to improve not only the individuals’ symptoms and functional outcomes, but also their overall quality of life (QOL). According to the World Health Organization (WHO), health-related quality of life (HRQOL) is defined as individuals’ perceptions of their living conditions related to goals, expectations, standards, and concerns within different cultures and value systems [1]. QOL assessments include subjective and objective measures. The former is preferable and centered as it focuses on the individuals’ particular perspectives and opinions, while the latter does not reflect their actual perception and life satisfaction [2]. Assessment of QOL can contribute to understand the main factors affecting individuals’ QOL and evaluate the efficacy of treatment strategies.

Schizophrenia is a chronic, enduring, and highly disabling psychotic disorder that typically begins in adolescence. In fact, many individuals exhibit non-specific subthreshold or transient psychotic symptoms such as suspiciousness, anxiety, and depressive symptoms accompanied by decreased social function before the onset of illness, referred to as the clinical high risk (CHR) state. High-risk individuals had to meet one or more of the prodromal syndrome criteria: brief intermitted psychotic symptoms syndrome (BIPS), attenuated psychotic symptoms syndrome (APSS), genetic risk and deterioration syndrome (GRD). Over one-third of individuals with CHR will progress to severe psychiatric disorders, mainly represented by schizophrenia, within the next 2 to 3 years [3], and nearly one-third of individuals who do not develop psychosis experience persistent functional impairment [4]. Their life satisfaction should be valued regardless of whether the individual converted into psychosis. The scale scores of subjective QOL for CHR have been proved to be lower than that of first-episode psychosis and healthy individuals, while the severity of symptoms and impairments of function and cognition falls somewhere between that of them [5–7]. Therefore, it is necessary to explore the influence of illness status/current symptom severity and other relevant factors on overall QOL.

Previous studies have shown that lower QOL scores in individuals at CHR for psychosis are associated with the severity of depressive/anxiety symptoms [8, 9], while associations with the severity of positive, negative, and depressive/anxiety symptoms have been reported in individuals with schizophrenia [6, 10], which may be attributed to the characteristics of the course of illness. Significant depressive symptoms or anxiety are present in the prodromal stage of psychosis [11]. After conversion to early psychosis, positive and negative symptoms are more pronounced. In addition, The percentage of individuals with schizophrenia who suffer from depressive symptoms is 27% to 31% [12]. Subjective life satisfaction is often inversely related to the heightened severity of depressive and anxiety symptoms, which tend to be more prominent in individuals with schizophrenia and those at CHR for psychosis. Nevertheless, there is promising evidence that psychotherapy and antidepressant therapy can play a crucial role in ameliorating these symptoms and enhancing overall QOL [13].

Cognitive impairment in schizophrenia, especially in aspects of executive function, attention, and memory, can affect social function and QOL indirectly [14, 15]. Currently, specific cognitive remediation therapies have been proposed, but their efficacy and impact on the QOL are not fully understood [16]. Other studies have shown that there is no correlation between QOL and cognitive impairment [17, 18]. Firm conclusions about specific cognitive domains associated with QOL cannot be drawn from the relevant literature as they rely on different tools for assessment. Besides, general demographic factors that affect the QOL include age, gender, marital status, education level, duration of illness, employment status, antipsychotic medications, etc. [15, 19–23], with results differing considerably among heterogeneous studies.

Particularly, there are limited reports directly comparing subjective QOL of FES and individuals at CHR for psychosis in China, and the reported factors of decided importance are unreliable for variety. We hypothesized that the QOL score is lower in individuals at CHR for psychosis than in those with FES, and related to depressive symptoms, negative symptoms, and unemployment. Therefore, the present study evaluated the degree of QOL impairment between individuals at CHR for psychosis and FES, along with their associated influencing factors.

## Methods

This study was reviewed and approved by the Ethics Committee of Beijing Anding Hospital Affiliated to Capital Medical University, and was conducted between January 2015 and January 2018 at Beijing Anding Hospital, Capital Medical University, Beijing, China. Individuals enrolled in the FES and CHR were inpatients or outpatients of this hospital, while those enrolled in the HC were recruited through advertising. All the individuals and their legal guardians were aware of the objective of this study and signed the informed consent form.

### Individuals

For inclusion, all individuals were aged between 14 and 40 years and educated beyond elementary school. Individuals with schizophrenia met the diagnostic criteria for FES designated by the Structured Clinical Interview for DSM-IV Axis I disorders-Patient Edition (SCID-I/*P*) [24]. In addition, they had illness duration of less than 3 years, no history of medication, or had not been on antipsychotics for more than 1 month since the onset. CHR individuals were screened through the Structured Interview for Psychosis Risk Syndromes (SIPS) to meet at least one of the three syndromes. BIPS: the individual’s positive symptoms have reached a psychotic level of intensity in the past three months and have occurred at a frequency of at least once per month, with each occurrence lasting for at least several minutes; APSS: the individuals exhibit subthreshold positive psychotic symptoms that have developed or worsened in the past year, occurring at least once a week in the past month; GRD: the individuals have a first-degree relative with a psychotic disorder and/or have met the criteria for DSM-5 Schizotypal Personality Disorder, along with experiencing a 30% or greater drop in the GAF score over the past year [25]. Healthy individuals without a history of psychiatric disorders and family history of such disorders were included. Potential individuals were excluded due to severe organic illness, or receiving modified electroconvulsive therapy 6 months before inclusion.

### Measures

#### Clinical assessment

Symptoms of the FES was assessed with the Positive and Negative Syndrome Scale (PANSS) [26]. The five-dimensional structure of the PANSS includes 9 items covering positive (negative) symptoms; 6 items concerning disorganized thoughts; and 4 items each regarding anxiety/depressive symptoms and impulsivity/hostility [27]. Each item has a defined and specific 7-level operational scoring standard (0–7). The scale was evaluated by trained psychiatrists, combining psychiatric examination, clinical examination, and relevant information provided by relatives. The evaluation was performed within one week of gathering the information, and lasted 30 to 50 min.

The clinical symptoms of individuals at CHR for psychosis were assessed using the scale of prodromal symptoms (SOPS) in SIPS [25]. There were 19 basic items, which were divided into four symptom subscales: positive (P1-P5); negative (N1-N6); disorganization (D1-D4); and general (G1-G4). Previous literature has shown that depressive symptoms significantly influence QOL, so dysphoric mood (G2) was also selected into the regression analysis. The symptoms were evaluated according to the occurrence, duration, frequency, intensity, and degree of conflict of symptoms. The scoring range for each item varies from 0 (no abnormality) to 6 (severe psychotic symptoms). The evaluator scored according to the definition and grading criteria of the SOPS items.

#### QOL assessment

Individuals’ QOL was assessed using the Chinese version of the WHOQOL-BREF [28], which contained 26 self-assessment items and produced scores in four domains: physical health, psychological health, social relationships, and environment (7, 6, 3, and 8 items respectively). It also included two items of independent analysis regarding individuals’ overall subjective feelings. All items were rated on a 5-point Likert scale, and mean scores for each domain were transformed into a 0-to-100 scale. The total score is the sum of four sub domains. Field scores were in the positive direction (i.e., the higher the score, the better the QOL).

#### Neurocognitive assessments

The Chinese version of the Simple Webster’s Adult Intelligence Test (IQ) [29] encompasses four sub-tests: knowledge, similarity, mapping, and block diagram tests. The MATRICS Consensus Cognitive Battery (MCCB) measures individuals’ neuropsychological state [30], including 7 cognitive domains: information processing speed; attention/alertness; working memory; verbal learning; visual learning; reasoning and problem solving; and social cognition.

### Statistical analysis

Data were analysed using the IBM SPSS Statistics 23.0 for Windows (SPSS, Chicago, IL, USA). One-way analysis of variance (*F*) was applied to process general demographic data, clinical data, and QOL total score. Multivariate analysis of variance was used to analyse the four sub-domains of QOL. Cognitive data were evaluated via covariance analysis (*F*), with age and intelligence quotient (IQ) as covariates. The Bonferroni method was employed for pairwise comparison. Categorical variables were analysed by chi-squared test (χ2). Associations among clinical characteristics, neurocognitive functions, and total QOL scores were analysed via Pearson’s correlation analysis for normally distributed data. Spearman’s rank correlation was used for data with non-normal distribution, and Bonferroni’s correction was applied for the significance level of the correlation coefficient for multiple comparisons. Stepwise multiple regression analysis was used to identify significant factors associated with individuals’ subjective QOL. A *P* value < 0.05 denoted a statistical significance.

## Results

### Demographic and clinical characteristics

During the enrollment process, 1 individual with FES and 1 individual at CHR for psychosis failed to complete the self-assessment of the QOL scale due to uncooperativeness. Thus, the final study population included 77 FES, 59 CHR and 64 HC, all of whom met the enrollment criteria for the respective group.

There were no significant differences in gender ratio, marital status, or education among the three groups (Table 1). The CHR group was significantly younger than the other 2 groups (*P* < 0.001). The rate of unemployment in the FES group was significantly higher than that of the CHR or HC group (*P* < 0.001). The proportion of individuals with family history of psychotic disorders in the CHR group was significantly higher than that in the FES group (*P* < 0.001). The majority (79%) of the FES group used antipsychotic drugs, compared to 27% in CHR group.

**Table 1:** Demographics and clinical features of the participants ^a^

	FES	CHR	HC	Total	*F*	*P*	Post hoc analysis
individuals, n	77	59	64	200	—	—	—
Age, y	25.4 ± 6.7	21.7 ± 5.4	25.2 ± 3.8	24.3 ± 5.7	9.07	< 0.001	CHR < FES, HC
Education, y	13.0 ± 3.3	13.2 ± 3.2	14.3 ± 3.4	13.4 ± 3.3	3.02	0.05	—
Duration of illness, mo	25.8 ± 25.8	23.2 ± 26.2	—	24.7 ± 25.9	0.34	0.56	—
IQ	99.7 ± 13.1	109.0 ± 11.6	113.3 ± 12.1	107.5 ± 13.5	17.90	< 0.001	FES < CHR, HC
SIPS							
Positive	—	9.4 ± 3.9	0.3 ± 1.2	4.6 ± 5.3	16.74	< 0.001	HC < CHR
Negative	—	9.4 ± 5.7	0.2 ± 0.8	4.5 ± 6.0	12.06	< 0.001	HC < CHR
Disorganization	—	4.7 ± 3.3	0.1 ± 0.5	2.3 ± 3.2	10.18	< 0.001	HC < CHR
General	—	5.4 ± 3.6	0.1 ± 0.5	2.6 ± 3.6	10.91	< 0.001	HC < CHR
Total score	—	28.8 ± 12.7	0.8 ± 2.6	13.9 ± 16.6	16.32	< 0.001	HC < CHR
PANSS							
Positive	31.1 ± 5.9	—	—	—	—	—	—
Negative	22.7 ± 8.5	—	—	—	—	—	—
Anxiety/depressive symptoms	7.3 ± 3.0	—	—	—	—	—	—
Impulsivity/hostility	11.5 ± 3.9	—	—	—	—	—	—
Disorganized thoughts	14.3 ± 4.0	—	—	—	—	—	—
Total score	85.4 ± 14.0	—	—	—	—	—	—
QOL							
Physical health	13.2 ± 2.6	12.8 ± 2.8	15.6 ± 1.9	13.8 ± 2.8	24.26	< 0.001	FES, CHR < HC
Psychological health	13.2 ± 2.4	12.2 ± 2.9	15.4 ± 1.8	13.6 ± 2.7	30.92	< 0.001	CHR < FES < HC
Social relationship	13.0 ± 3.1	12.2 ± 2.9	14.9 ± 2.2	13.4 ± 3.0	16.06	< 0.001	FES, CHR < HC
Environment	13.3 ± 2.2	13.3 ± 2.1	14.1 ± 2.1	13.5 ± 2.2	3.22	0.042	FES, CHR < HC
Total score	73.9 ± 15.2	66.3 ± 19.1	84.2 ± 10.1	75.0 ± 16.6	21.55	< 0.001	CHR < FES < HC
MCCB Domains							
Processing speed	33.0 ± 8.7	38.5 ± 7.5	45.2 ± 6.9	39.0 ± 9.2	23.36	< 0.001	FES < CHR < HC
Attention/Vigilance	29.9 ± 9.9	39.8 ± 11.3	46.0 ± 8.5	38.8 ± 11.9	26.80	< 0.001	FES < CHR < HC
Working memory	38.3 ± 10.0	37.2 ± 14.7	46.6 ± 6.9	40.9 ± 11.6	7.47	0.001	FES, CHR < HC
Verbal learning	38.5 ± 8.9	42.6 ± 9.7	47.6 ± 8.8	42.9 ± 9.9	7.01	0.001	FES, CHR < HC
Visual learning	39.4 ± 13.8	42.6 ± 11.2	47.1 ± 10.3	43.1 ± 12.2	1.17	0.31	—
Reasoning/problem solving	34.5 ± 11.0	39.9 ± 11.3	43.4 ± 10.5	39.3 ± 11.5	2.62	0.07	—
Social recognition	31.9 ± 12.2	37.4 ± 8.3	39.3 ± 9.8	36.1 ± 10.7	6.12	0.003	FES < CHR, HC
Total score	35.5 ± 6.3	40.1 ± 5.9	45.0 ± 5.7	40.4 ± 7.2	21.69	< 0.001	FES < CHR < HC
					χ^2^	*P*	
Men	37 (48.1)	35 (59.3)	37 (57.8)	109 (54.5)	2.13	0.34	—
Married	16 (20.8)	5 (8.5)	11 (17.2)	32 (16)	3.86	0.15	—
Family history	15 (19.5)	18 (30.5)	—	33 (16.5)	21.93	< 0.001	FES < CHR
Smoking	7 (9.1)	6 (10.2)	7 (10.9)	20 (10)	0.14	0.94	—
Unemployed	32 (41.6)	7 (11.9)	4 (6.3)	43 (21.5)	30.42	< 0.001	CHR, HC < FES
Medication	67 (87.0)	34 (57.6)	—	101	—	—	—
Unmedicated	10 (13.0)	25 (42.4)	—	35	—	—	—
AP	61 (79.2)	16 (27.1)	—	77	—	—	—
AD	0	8 (13.5)	—	8	—	—	—
AD + AP	1 (1.3)	6 (10.2)	—	7	—	—	—
Unspecified	5 (6.5)	3 (5.1)	—	8	—	—	—
TCM	0	1 (1.7)	—	1	—	—	—

*AD* Antidepressant, *AP* Antipsychotic, *TCM* Traditional Chinese medicine

^a^ Data are reported as n (%), unless indicated otherwise

The IQ scores was highest in the HC group and was lowest in the FES group (*P* < 0.001; Table 1). The PANSS scale indicated that the FES group had obvious psychotic symptoms, and the positive symptom scores of the CHR group were significantly higher than those of the HC group (*P* < 0.001). Regarding the scores of the cognitive total domain and the sub-domain (except visual learning and reasoning/problem solving), the FES and CHR groups were lower than the HC group. The order of groups by total MCCB score, from high to low, was HC, CHR, and FES groups.

### Group comparisons of QOL

One-way ANOVA showed significant differences among the three groups in total QOL scores (*F* = 21.55, *P* < 0.001); The order of groups by total QOL scores, from high to low, were HC, FES, and CHR group (Table 1). The multivariate analysis of variance revealed that the group differences were statistically significant for physical health (*F* = 24.26, *P* < 0.001), psychological health (*F* = 30.92, *P* < 0.001), social relationship (*F* = 16.06, *P* < 0.001), and environment (*F* = 3.22, *P* = 0.042).

**Fig. 1:**
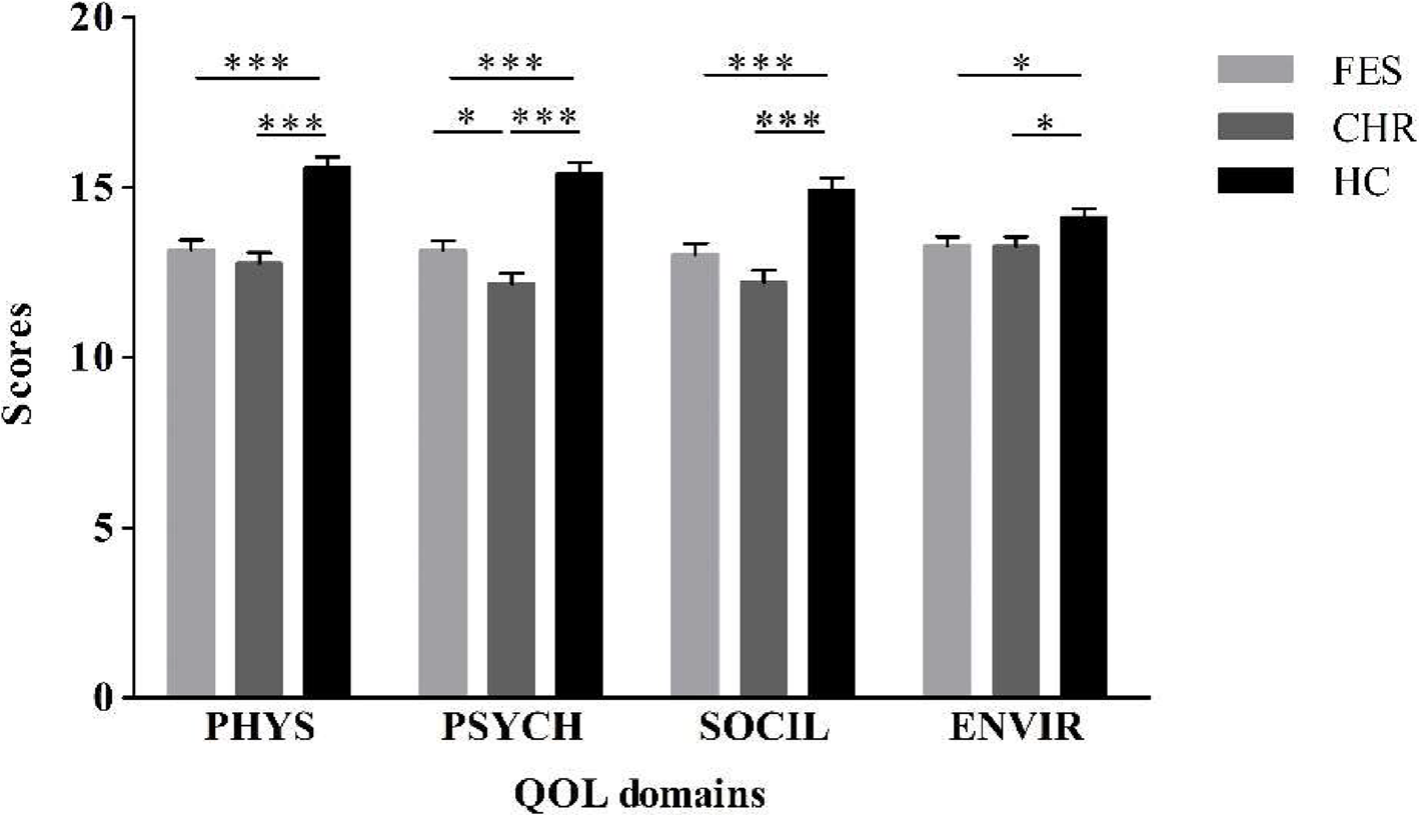
Comparison of WHOQOL-BREF domain scores of the FES, CHR, and HC groups. Abbreviations: PHYS, physical health; PSYCH, psychological health; SOCIL, social relationship; ENVIR: environment. *P < 0.05, **P < 0.01, ***P < 0.001

Compared with the HC group, the scores of the FES and CHR groups were lower in the total WHOQOL-BREF and four sub-domains (Fig. 1). In terms of the total WHOQOL-BREF and psychological health scores, the CHR group was lower than the FES group.

### Variables associated with QOL in the FES and CHR groups

According to the Pearson’s or Spearman’s correlation analyses, in the FES group, the total QOL score was negatively correlated with the severity of anxiety/depressive symptoms (*r* = –0.34, *P* = 0.003; Table 2). In the CHR group, the total QOL score was inversely correlated with the severity of negative (*r* = –0.29, *P* = 0.03) and dysphoric mood (*r* = –0.33, *P* = 0.01) symptoms. After adjustment for multiple comparisons with Bonferroni’s correction, the correlation between anxiety/depressive symptoms and total QOL scores was significant only in the FES group.

**Table 2:** Correlations between clinical characteristics, neurocognition and QOL scores in FES and CHR

	FES		CHR	
	*r*	*P*	*r*	*P*
PANSS				
Positive	–0.01	0.91	—	—
Negative	–0.03	0.83	—	—
Anxiety/depressive symptom	–0.34	0.003 *	—	—
Impulsivity/hostility	–0.10	0.41	—	—
Disorganized thoughts	–0.13	0.26	—	—
SIPS				
Positive	—	—	–0.24	0.08
Negative	—	—	–0.29	**0.03**
Disorganization	—	—	–0.24	0.08
Dysphoric mood	—	—	–0.33	**0.01**
MCCB total score	–0.017	0.91	0.14	0.40

^*^ Significant after Bonferroni’s correction for multiple comparisons [11 comparisons; *P* < 0.05/11]

Based on the multiple linear regression of demographic, clinical symptoms, and cognition of the QOL domains (Table 3), in the FES group, anxiety/depressive symptoms had negative effects on the psychological health (*t* = –3.34, *P* = 0.002) and total (*t* = –3.93, *P* < 0.001) scores of the QOL. Unemployment negatively influenced physical health (*t* = –2.16, *P* = 0.036) and social relationship (*t* = –3.20, *P* = 0.003). Impulsivity/hostility showed negative effects on physical health (*t* = –2.28, *P* = 0.028) and psychological health (*t* = –2.71, *P* = 0.01). In addition, the positive influence of negative symptoms on the social relationship domain of QOL was found (*t* = 2.95, *P* = 0.005).

**Table 3:** Multivariate regression analysis of influential factors on the life qualities of FES and CHR

	Dependent variable	Independent variable	*R*^2^	Beta	*t*	*P*
FES	Physical health	Unemployed	0.14	–0.30	–2.16	0.036
		PANSS-Impulsivity/hostility		–0.31	–2.28	0.028
	Psychological health	PANSS-Anxiety/depressive	0.29	–0.42	–3.34	0.002
		PANSS-Impulsivity/hostility		–0.34	–2.71	0.01
	Social relationship	Unemployed	0.22	–0.44	–3.20	0.003
		PANSS-Negative		0.40	2.95	0.005
	QOL total score	PANSS-Anxiety/depressive	0.24	–0.51	–3.93	< 0.001
CHR	Physical health	SIPS-Disorganization	0.42	–0.32	–2.49	0.018
		Unemployed		–0.72	–5.10	< 0.001
		Age		0.34	2.41	0.022
	Psychological health	SIPS-Negative	0.21	–0.49	–3.32	0.002
	Social relationship	Unemployed	0.10	–0.35	–2.23	0.032
	Environment	SIPS-dysphoric mood	0.19	–0.35	–2.93	0.005
		MCCB total score		0.28	2.35	0.023
	QOL total score	SIPS-Disorganization	0.31	–0.46	–3.34	0.002
		Unemployed		–0.46	–2.97	0.005
		Age		0.33	2.14	0.04

In the CHR group, unemployment had a negative influence on physical health (*t* = –5.10, *P* < 0.001), social relationship (*t* = –2.23, *P* = 0.03), and the overall domain (*t* = –2.97, *P* = 0.005). The overall QOL score and physical health improved with age. Moreover, the effects of dysphoric mood, disorganization symptoms, negative symptoms, and cognitive impairment on QOL were found.

## Discussion

This cross-sectional study investigated the cognition and QOL of individuals with FES or CHR versus HC. The main findings were, first, that individuals with FES and CHR demonstrated severe impairment of cognition and degraded QOL relative to HC. Furthermore, the total QOL score and psychological health score of CHR individuals were significantly lower than those of FES. Secondly, the QOL of individuals with FES was principally affected by unemployment and clinical symptoms, with anxiety/depressive symptoms being the most prominent factor, as indicated by the correlation and multiple regression analyses for the overall QOL and subdomains. The QOL of CHR individuals was influenced most significantly by unemployment, but also age, negative symptoms, disorganization, dysphoric mood, and neurocognition, indicated by the QOL overall and subdomains.

Reviewing the existing literature on predicting psychosis, there are differences among studies in selecting criteria for CHR individuals. It is not yet clear to what extent these differences lead to varying conversion rates among CHR individuals. However, existing research consistently suggests that CHR diagnostic criteria are more practical in detecting individuals at an increased risk of developing psychosis [31]. In the identification of CHR individuals and the differentiation of various types of risk syndromes, researchers typically use two main diagnostic tools: the Comprehensive Assessment of At-Risk Mental States (CAARMS) and the Structured Interview for Prodromal Syndromes (SIPS) [32]. Although these two tools have slight differences in diagnostic criteria, studies indicate that CHR individuals identified using both tools experience impairments in their quality of life, and these impairments are more severe than those observed in individuals experiencing their first episode of psychosis [6, 8, 33, 34]. This study has also found similar research results. Compared to individuals with psychiatric disorders, individuals at CHR for psychosis reported more stress, mental vulnerability, lack of self-confidence, and poor social adaptability. They are dissatisfied with their social relationships and expressed dissatisfaction with their overall life [35].

A number of studies of schizophrenia and CHR have shown a significant association between anxiety/depressive symptoms and QOL [8, 36–40]. In a follow-up study of CHR, improvement of the QOL score was associated with alleviation of depressive symptoms over time [8]. Depressive symptoms might be inherent to the CHR state and the expression of an early, mild stage of the same neurobiological process that causes psychosis [41]. Consistent with previous studies, this study found that anxiety/depressive symptoms of individuals with FES negatively influenced the psychological health and total QOL scores, dysphoric mood of individuals with CHR negatively influenced the environmental field. Individuals with depressive symptoms appear to be more aware of their illness consequences. Depressive cognitions, such as self-deprecation and feelings of hopelessness, may influence individuals’ subjective assessments of life quality [42]. Moreover, cognitive behavioral therapy or antidepressants can relieve depressive symptoms and enhance their life satisfaction [6, 13]. Therefore, interventions for depressive symptoms in CHR and FES individuals should also be given due attention.

The present study indicated that lower scores in psychological health were associated with the severity of negative symptoms among individuals with CHR. Previous studies have also reported that degraded QOL was associated with lower functional abilities and negative symptoms, which affect social relations, self-esteem, positive and negative feelings, learning, and memory [10, 15]. Additionally, negative symptoms of individuals with FES were positively associated with social relationship of QOL in this study, contrary to previous research conclusions [5, 10, 15, 43], which required more replication to be known for sure. The above equivocal results might stem from the subjective measurement method [44]. An effect from positive symptoms on QOL of individuals with FES has not been found. Disorganization symptoms of CHR individuals showed a significant association with physical health and total QOL score, which is consistent with the results of cross-sectional and longitudinal follow-up studies [8, 44]. Indeed, negative symptoms are superior to positive symptoms in predicting poor QOL [10].

In the present study, no correlation was found between QOL and various domains of neurocognition. In the regression analysis, only the MCCB total score of CHR individuals could predict the QOL environmental field. Some studies have found that QOL is related to information processing speed, memory, and executive function [15, 45–47], but others reported negative results [18, 48]. Social cognition is more predictive of functional outcomes in schizophrenia than neurocognition, which may be a factor affecting QOL [49]. But no correlation between the two was found in this study. Meta analysis also showed that neurocognition was positively correlated with objective QOL in schizophrenia, but not with subjective QOL [50]. Theoretically, neurocognition and its associated symptoms affect social function, which further influences the QOL [46]. However, it must be considered that only one item in the WHOQOL-BREF scale is specifically for cognitive function. If cognitive function is given more weight on the QOL questionnaire, the findings may align more closely [45].

The present study found that unemployment of individuals with FES was associated with the aspects of physical health and social relationship in the subjective QOL. Unemployment of individuals with CHR was also associated with the above two domains and the overall QOL score. Employment not only provides economic remuneration but also improves the QOL and social function of patients and enhances their self-esteem and self-confidence, playing an important role in the rehabilitation of individuals with psychiatric disorders. Unemployment seems to induce perceived stress, leading to a decrease in QOL, especially among FES or CHR individuals with poor social adaptability. Individuals’ rehabilitation is improved when individuals are encouraged to work and social prejudice against mental illness is eliminated [21, 51].

Consistent with previous findings, the multiple regression analysis of the present study indicated that, for individuals with CHR, age was positively associated with QOL [15, 52]. Several explanations are proposed. First, younger individuals are more concerned about the effect of mental symptoms on their studies and interpersonal associations, while the older ones worry less about material wealth and family life, and may have lowered their expectations when living with the illness in order to adapt to the situation [52]. It is also possible that poor insight leads to false self-report of QOL [53, 54]. The specific reasons for QOL differences among the age groups in these individuals remain unclear, and further research and detailed evaluations are warranted.

This study has several limitations. Firstly, the depressive symptoms of individuals with FES or CHR were not explored thoroughly, and a dedicated depressive symptoms scale was not used for evaluation. Secondly, as there were no follow-ups for CHR individuals, it remains unclear concerning the percentage of individuals with CHR who developed psychosis subsequently. It is not possible to perform more detailed risk stratification for the CHR individuals at baseline, which restrict further subgroup analysis. Thirdly, three syndromes of CHR may have different risks of developing schizophrenia. Due to the smaller sample sizes of the BLIPS and GRD syndromes, stratified analyses between the subgroups cannot be conducted. Fourthly, 79.2% of individuals with FES and 27.1% of individuals with CHR had a history of antipsychotic drug use at the time of enrollment. Antipsychotic drugs may cause various adverse reactions while improving symptoms, affecting the assessment of their subjective QOL [20]. However, none of them has been evaluated in detail, so the relationship between antipsychotic drug use and QOL cannot be derived. Finally, this study mainly focuses on the measurement of QOL using the WHOQOL-BREF, i.e. self-reported/subjective QOL, without considering the functional/objective QOL metrics.

## Conclusions

Individuals at CHR for psychosis exhibited poorer subjective QOL compared to those of FES, especially in the psychological health domain. The influencing factors of QOL may be similar in the different illness stages of psychosis. Anxiety/depressive symptoms and social functioning may be key factors related to poorer QOL.

## Data Availability

Data are available from the first and the corresponding authors.
